# Impact of cardiovascular risk profile on COVID-19 outcome. A meta-analysis

**DOI:** 10.1371/journal.pone.0237131

**Published:** 2020-08-14

**Authors:** Jolanda Sabatino, Salvatore De Rosa, Giovanni Di Salvo, Ciro Indolfi

**Affiliations:** 1 Division of Cardiology, Department of Medical and Surgical Sciences, "Magna Graecia" University, Catanzaro, Italy; 2 Cardiovascular Research Center, "Magna Graecia" University, Catanzaro, Italy; 3 Department of Women's and Children's Health, University of Padua, Padua, Italy; Campus Bio-Medico University of Rome, ITALY

## Abstract

**Background:**

The ongoing pandemic of *Novel Coronavirus Disease 2019* (COVID-19) infection has created a global emergency. Despite the infection causes a mild illness to most people, some patients are severely affected, demanding an urgent need to better understand how to risk-stratify infected subjects.

**Design:**

This is a meta-analysis of observational studies evaluating cardiovascular (CV) complications in hospitalized COVID-19 patients and the impact of cardiovascular risk factors (RF) or comorbidities on mortality.

**Methods:**

Data sources: PubMed, Scopus, and ISI from 1 December 2019 through 11 June 2020; references of eligible studies; scientific session abstracts; cardiology web sites. We selected studies reporting clinical outcomes of hospitalized patients with COVID-19. The main outcome was death. Secondary outcomes were cardiovascular symptoms and cardiovascular events developed during the COVID-19-related hospitalization. Extracted data were recorded in excel worksheets and analysed using statistical software (MedCalc, OpenMetanalyst, R). We used the proportion with 95% CI as the summary measure. A Freeman-Tukey transformation was used to calculate the weighted summary proportion under the random-effects model. Heterogeneity was assessed by using the Cochran Q test and I^2^ values.

**Results:**

Among 77317 hospitalized patients from 21 studies, 12.86% had cardiovascular comorbidities or RF. Cardiovascular complications were registered in 14.09% of cases during hospitalization. At meta-regression analysis, pre-existing cardiovascular comorbidities or RF were significantly associated to cardiovascular complications in COVID-19 patients (p = 0.019). Pre-existing cardiovascular comorbidities or RF (p<0.001), older age (p<0.001), and the development of cardiovascular complications during the hospitalization (p = 0.038) had a significant interaction with death.

**Conclusions:**

Cardiovascular complications are frequent among COVID-19 patients, and might contribute to adverse clinical events and mortality, together with pre-existing cardiovascular comorbidities and RF. Clinicians worldwide should be aware of this association, to identifying patients at higher risk.

## Introduction

Since December 2019 a new epidemic outbreak has emerged from China, creating an alert worldwide (1). The underlying pathogen was confirmed, through a genomic sequencing, to be a new positive-sense, single-stranded RNA coronavirus, named severe acute respiratory syndrome coronavirus 2 (SARS-CoV-2), which did not match any previous known virus [1–4]. While a growing number of cases are being confirmed globally, SARS-CoV-2 appears to have greater infectivity and a lower-case fatality rate when compared to previous SARS and MERS [5].

For most people the Novel Coronavirus Disease 2019 (COVID-19) has caused mild illness, however, it can generate severe pneumonia in some patient, and it can be fatal in others [2–4, 6–9]. Older people, and those with pre-existing medical conditions (such as cardiovascular disease, chronic respiratory disease or diabetes) are at risk for severe disease. However, it is crucial for clinicians working with cardiovascular patients to understand clinical presentation, natural history and risk factors for COVID-19 infection. Therefore, this study characterizes the cardiovascular (CV) risk profile of COVID-19 patients, along with the CV complications developed during hospitalization and investigates their impact on case fatality rates.

## Methods

The protocol for this meta-analysis was developed before performing the analyses and registered in PROSPERO (http://www.york.ac.uk/inst/crd; CRD42020191650).

### Data sources and study selection

We searched PubMed, Scopus, and Google Scholar electronic databases from 1 November 2019 through 11 June 2020 using the following keywords: coronavirus, Covid-19, cardiac, cardiovascular. The following search terms were used: i) PubMed MEDLINE—(("COVID-19"[All Fields] OR "severe acute respiratory syndrome coronavirus 2"[Supplementary Concept] OR "severe acute respiratory syndrome coronavirus 2"[All Fields] OR "2019-nCoV"[All Fields]) OR sars-cov2[All Fields]) AND (("cardiovascular system"[MeSH Terms] OR ("cardiovascular"[All Fields] AND "system"[All Fields]) OR "cardiovascular system"[All Fields] OR "cardiovascular"[All Fields]) OR ("heart"[MeSH Terms] OR "heart"[All Fields] OR "cardiac"[All Fields]) OR CVD[All Fields] OR CRF[All Fields] OR ("blood vessels"[MeSH Terms] OR ("blood"[All Fields] AND "vessels"[All Fields]) OR "blood vessels"[All Fields] OR "vascular"[All Fields])); ii) SCOPUS—covid-19 OR sars-cov-2 AND cardiovascular; iii) ISI Web of Science—covid-19, cardiovascular disease. We also checked the reference lists of eligible studies and screened scientific abstracts and relevant Web sites (www.who.int; www.escardio.org; www.medscape.com, www.acc.org).

Two investigators (J.S and S.D.R.) independently screened search records to identify eligible studies. No disagreements occurred. Inclusion criteria were clinical studies describing patients with Covid-19 and CV comorbidities and/or developing CV complications. Exclusion criteria were duplicate publications and studies in which the cardiovascular comorbidities or complications were not specified. Studies in Chinese directly resulting from database search were translated to English before analysis. Non-english-language citations were excluded. Pre-print manuscripts were allowed to warrant inclusion of the largest number of studies available. In case of lacking data, the corresponding authors were contacted by email to request additional details. Inclusion and exclusion criteria are summarized according to the PICOS approach in Table 1.

**Table 1:** Inclusion and exclusion criteria using the PICOS approach.

	Item	Description	Inclusion Criteria	Exclusion Criteria
**P**	**Population**	Hospitalized patients with COVID-19	Hospitalized patients with COVID-19 and in-hospital outcome reported (at least mortality available). Cardiovascular comorbidities and risk factors reported. Cardiovascular complications reported	Studies presenting data already published within another study already included (in case of duplicity, the largest study reporting the data required will be selected). Hospitalization place or time not reported.
**I**	**Intervention**	No actual intervention. Studies need to have included hospitalized patients and report in-hospital mortality	Hospitalized patients.	Community- and epidemiology reports of non-hospitalized patients.
**C**	**Comparison**	Pre-specified subgroup analysis: patients managed in the Intensive Care Unit vs. Non-intensive Care Wards.	Description of care type.	Intensity of care not specified.
**O**	**Outcome**	Primary: all-cause death Secondary: Cardiovascular Complications	In-hospital outcome data reported. Cardiovascular comorbidities and risk factors reported. Cardiovascular complications reported.	In-hospital death not reported.
**S**	**Study design**	Retrospective and case series	Retrospective studies. Case series. Research letters. Pre-prints.	Letters to the editor. Guidelines. Review articles. In vitro pharmacological studies. Animal studies.

### Data extraction and quality assessment

Two reviewers (JS and SDR) independently extracted data about study characteristics and event rates from full articles. Two investigators (JS and SDR) independently assessed study quality for each study. The assessment was done at the study level and focused on the main study outcome (CV comorbidities and/or complications in patients with Covid-19). Disagreements were resolved by consensus. The following data were extracted: total number of patients; age; gender; geographic location; enrolment period; cardiovascular risk profile; pre-existing cardiovascular disease; in-hospital events (death, cardiovascular death, AMI, myocardial injury, cerebrovascular accidents, acute heart failure). Confounding factors were independently assessed by two investigators (JS and SDR). Site and time interval of patient inclusion was assessed for all eligible studies to avoid duplicity of data. In case of overlap between the studies, the largest study including the required information was selected over the other(s).

Quality assessment was performed according to the Quality Assessment Tool for Observational Cohort and Cross-Sectional Studies (QAT-OC/CSS; https://www.nhlbi.nih.gov/health-topics/study-quality-assessment-tools) of the National Institute of Health (NIH, Bethesda, MD, USA) (S1 Table).

### Data synthesis and analysis

We based our primary analyses on CV comorbidities and/or complications data in patients affected by COVID-19. We used the proportion with 95% CI as the summary measure. A Freeman-Tukey transformation (arcsine square root transformation) was used to calculate the weighted summary proportion under the random-effects model, as previously described [10]. Heterogeneity was assessed by using the Cochran Q test by means of a chi-square function. P values below 0.10 were considered indicative of heterogeneity. I^2^ values were calculated to estimate variation among studies attributable to heterogeneity. Metanalysis results were displayed with forest plots in which the measure of effect for each study is represented by a square and the area of each square is proportional to study weight. Sensitivity analyses were performed in case of significant heterogeneity to assess the potential impact on the results. Arcsine of square root proportion (ASRP) with correction for continuity when a group had zero events was used as the metric for subgroup meta-analysis, as already previously described [11]. A meta-regression analysis was performed to examine the potential effect of age, cardiovascular comorbidities or cardiovascular risk factors on cardiovascular complications and death. Publication bias was assessed using Funnel plots. Analyses were performed by using MedCalc 15.8 and Open Meta-Analyst and R (The R Foundation).

## Results

**Fig 1:**
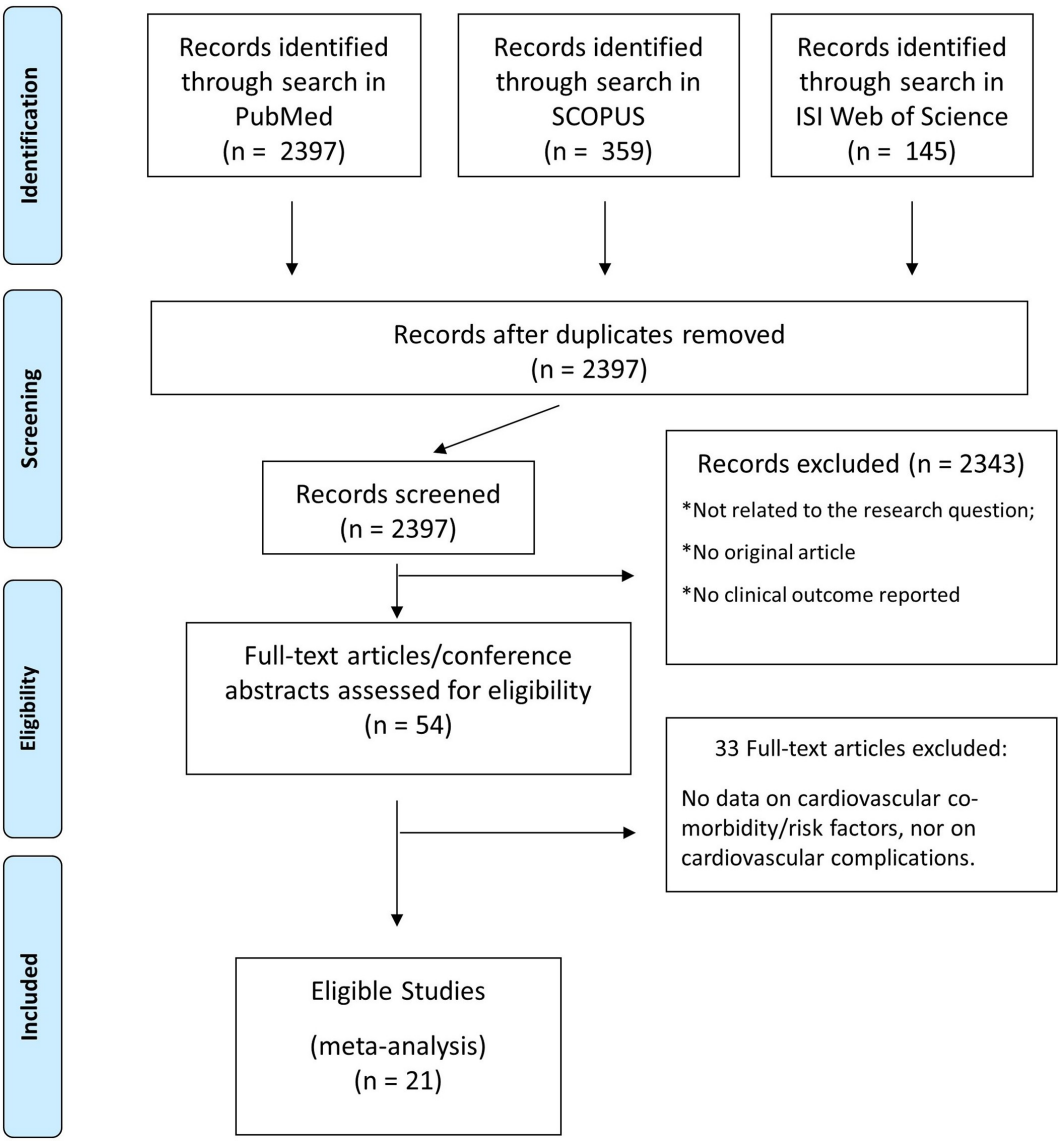
Study search and selection. PRISMA flowchart describing article search, screening and selection.

Of 2397 screened records, we identified 21 eligible studies that reported clinical outcomes of patients admitted to the hospital and with a confirmed positivity to the SARS-CoV-2 virus (Fig 1) [2–4, 6–9, 12–21]. To avoid duplicity of data, all eligible studies were checked for site and time interval of patient inclusion for every individual analysis. All studies included hospitalized patients, from different geographic areas, including Asia, Europe and the United States. Altogether, clinical data about 77317 hospitalized COVID-19 patients are reported within the studies selected (Table 2). The overall quality rating of the included studies was judged as “fair”, according to the Quality Assessment Tool for Observational Cohort and Cross-Sectional Studies of the NIH (S1 Table). In particular, all studies clearly state their objective- The study population was clearly specified in 20 studies with less specific definition of inclusion criteria in only one study among those included. The participation rate of eligible patient was very high across all studies. Outcome measures were generally clearly defined.

**Table 2:** Main characteristics of the selected studies.

Study	Recruitment Period	N	Age*	Sex^#^	Place
*Liu K*	30/12/19–24/01/20	137	57	76	***China***—Wuhan, Xiaogan, Yichang, Jingzou, Enshi Tujia and Miao, Shiyan
*Huang C*	16/12/19–02/01/20	41	49	11	***China***—Wuhan (Jin Yin-tan Hospital)
*Chen N*	01/01/20–20/01/20	99	55.5	32	***China***—Wuhan (Jin Yin-tan Hospital)
*Wang D*	01/01/20–28/01/20	138	56	63	***China***—Wuhan (Zhongnan Hospital)
*Ji D*	20/01/20–16/02/20	49	43.6	18	***China***—Wuhan (Fifth Medical Center of Chinese PLA General Hospital)
*Wu J*	Jan-2020—Feb 2020	80	44	38	***China***—Chongqing
*Zhang JJ*	16/01/20–03/02/20	140	57	69	***China***—Wuhan (Number 7 Hospital)
*Yang X*	24/12/19–26/01/20	52	59.7	17	***China***—Wuhan (Jin Yin-tan hospital)
*Peng YD*	20/01/20–15/02/20	112	62	59	***China***—Wuhan Union Medical Center
*Guan W*	11/12/19–29/01/20	1099	47	459	***China***—Medical Treatment Expert Group for Covid-19 (552 Hospitals in 30 provinces)
*China CDC*	31/12/19–11/02/20	44672	45.9	21691	***China*** CDC
*Goyal P*	3/03/20–27/03/20	393	62.2	155	***U*.*S*.**—NY Presbiterian + New York Cornell (2 Hospitals)
*Richardson S*	1/03/20–4/04/20	5700	63	2263	***U*.*S*.**—NYC + Long Island + Werchester C (12 Hospitals)
*Lala A*	27/02/20–12/04/20	2736	66.4	1106	***U*.*S*.*–****NYC Mount Sinai (5 Hospitals)*
*Garg S*	1/03/20–30/03/20	180	N/A	82	***U*.*S*.**
*Grasselli G*	20/02/20–18/03/20	1591	63	287	***Italy***—ICU Network Lombardy
*Kim ES*	19/01/20–17/02/20	28	42.6	13	***Korea***—National Committee
*Sun Y*	26/01/20–16/02/20	54	42	25	***Singapore***—National Center for Infectious Disease
*Buckner FS*	02/03/20–26/03/20	105	69	52	***U*.*S*.*–****Seattle (UW Medical Center)*
*Galloway JB*	01/03/20–17/04//20	1157	71	491	***U*.*K*.*–****London (King’s College and Princess Royal Hospitals)*
*Janbabaei G*	24/2/20–29/3/20	18754	55.2	7314	***IRAN*** *(31 provinces*, *879 hospitals)*

N = Number

* = mean age in years

# = number of females in the study; NYC = New York City; CDC = Center for Disease Control; ICU = Intensive Care Unit; U.S. = United States of America; U.K. = United Kingdom of Great Britain and Northern Ireland.

**Fig 2:**
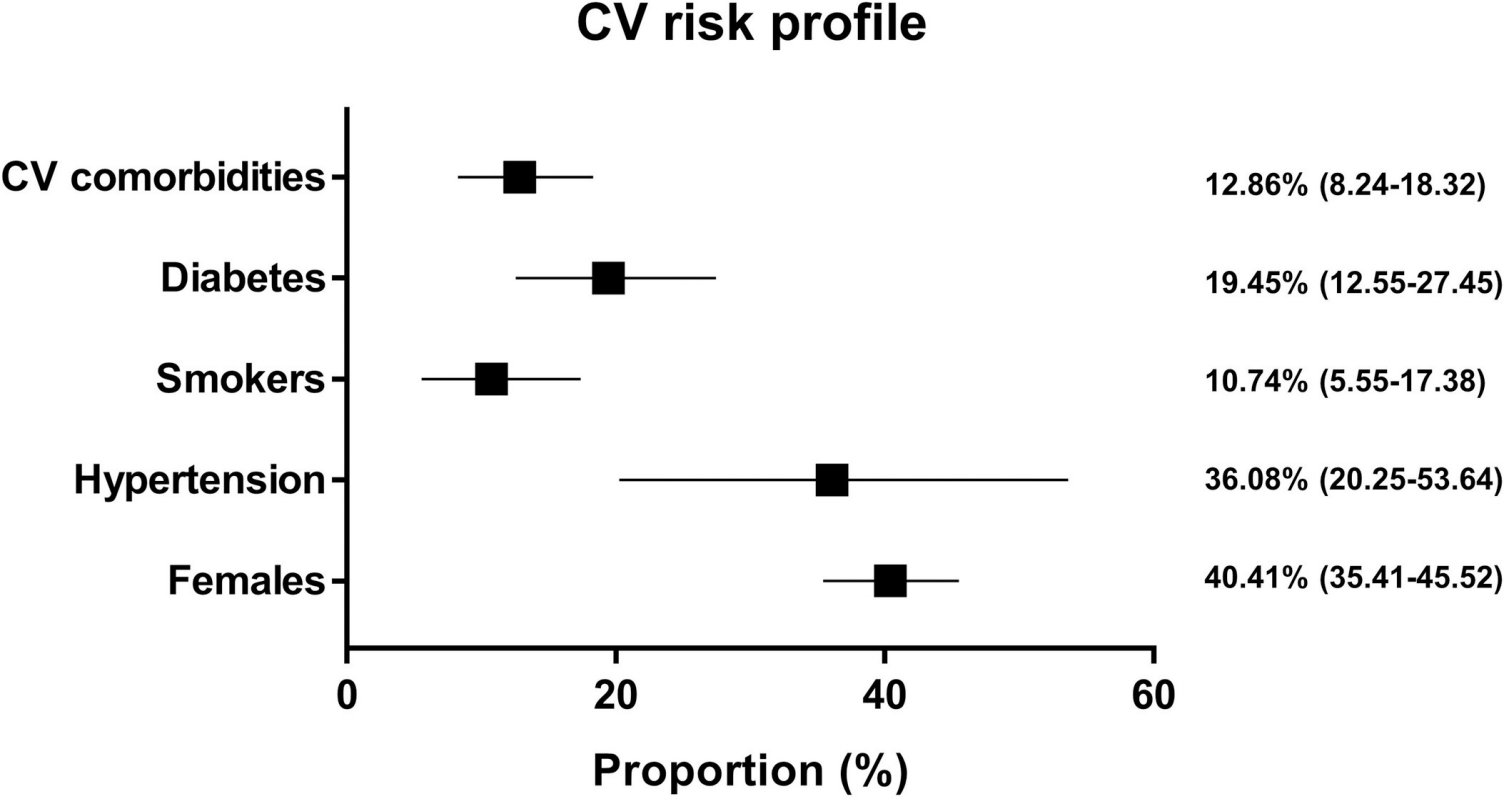
Cardiovascular risk profile of hospitalized COVID-19 patients. Each line represents the result of the meta-analysis for a single endpoint. The square represents the summary effect size (proportion) and the horizontal line the relative 95% Confidence Interval.

Overall, 40.41% (95% CI = 35.41–45.52) of the hospitalized patients (n = 77317) were females (Fig 2). Mean age was 48.4±18.5. Of all included patients, 12.89% (95% CI = 8.24–18.32) had any cardiovascular comorbidities (Fig 2), with large heterogeneity among the studies (I^2^ = 99.6%; Q = 1814; p<0.001). Specifically, hypertension was present in 36.08% (95% CI = 20.25–53.64) of all patients (Fig 2). In addition, 19.45% (95% CI = 12.55–27.45) of all patients suffered from diabetes (Fig 2). Moreover, 10.74% (95% CI = 5.55–17.38) of patients were smokers (Fig 1). Obesity was present at anamnesis in 33.78% (95% CI = 29.25–38.46) of patients. A diagnosis of coronary artery disease (CAD) was already known at presentation for 11.67% (95% CI = 7.34–16.84) of patients. Heart failure (HF) was present at anamnesis in 9.35% (95% CI = 6.19–13.09) of patients. A history of chronic obstructive pulmonary disease (COPD) was present for 5.30% (95% CI = 2.53–9.03) of patients (S1 Fig and S2 Fig). Funnel plots did not show the presence of a publication bias (S3 Fig and S4 Fig).

**Fig 3:**
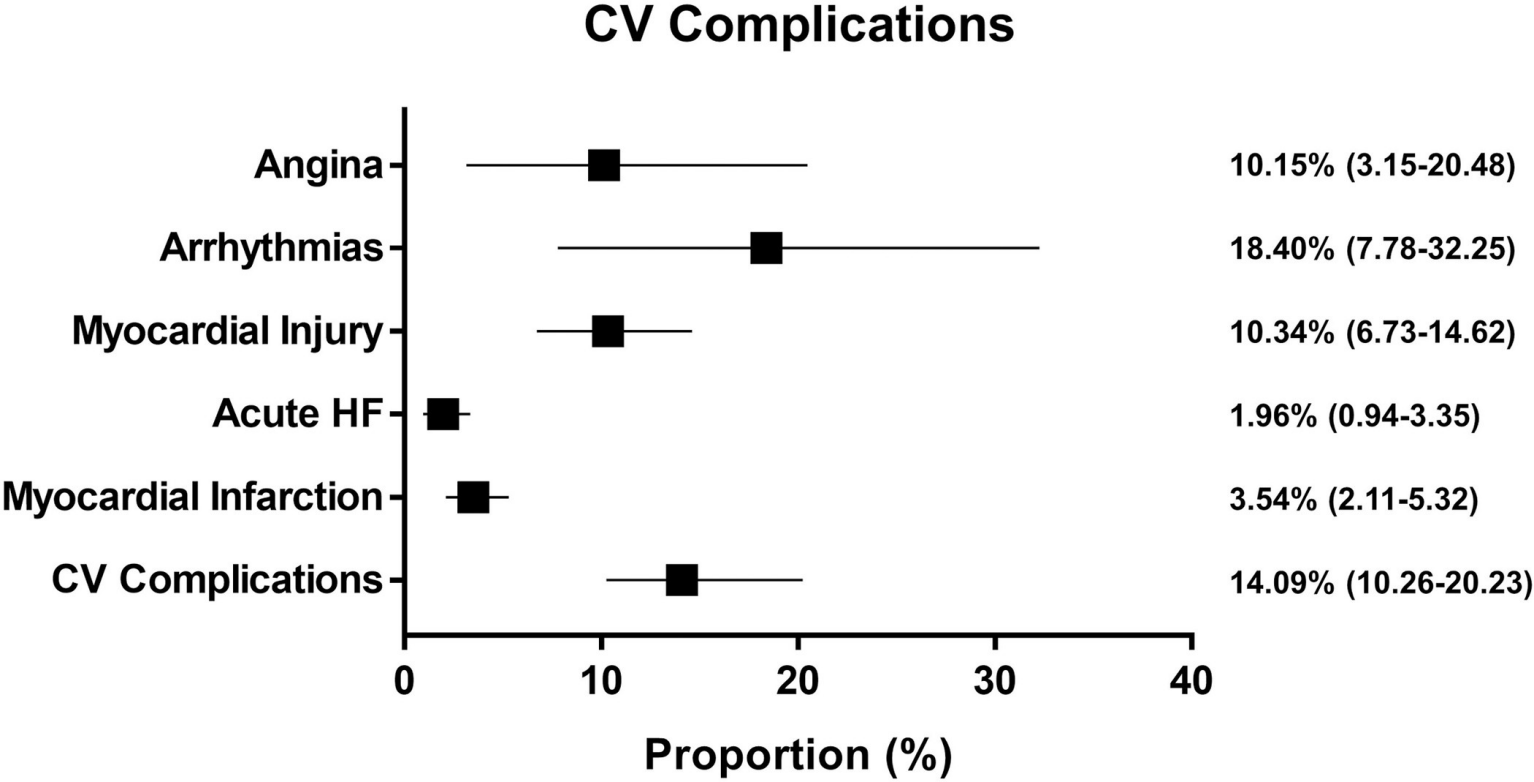
Cardiovascular complications in hospitalized COVID-19 patients. Each line represents the result of the meta-analysis for a single endpoint. The square represents the summary effect size (proportion) and the horizontal line the relative 95% Confidence Interval.

Cardiovascular complications were registered in 14.09% (95% CI = 10.26–20.23) (Fig 3). In particular, myocardial injury was documented in 10.34% (95% CI = 6.73–14.62) of patients. Angina was reported from 10.15% (95% CI = 3.16–20.48) of patients. Arrhythmias or palpitations were reported in 18.40% (95% CI = 7.78–32.25) of patients. Acute Heart Failure was reported in 1.96% (95% CI = 0.94–3.35) and acute Myocardial Infarction in 3.54% (95% CI = 2.11–5.32) of patients (S5 Fig). The Funnel plot did not show the presence of a publication bias (S6 Fig).

At meta-regression analysis, pre-existing cardiovascular comorbidities or risk factors were significant predictors of cardiovascular complications in COVID-19 patients (p = 0.026), while neither age (p = 0.097) nor gender (p = 0.224) were (Table 3).

**Table 3:** Predictors of cardiovascular complications at meta-regression analysis.

Covariate	Regression Coefficient	95% Confidence Interval	P value
Age	0.009	-0.005–0.023	0.197
Gender	-0.011	-0.026–0.005	0.173
CV comorbidity/risk factor	0.005	0.001–0.009	0.019

**Fig 4:**
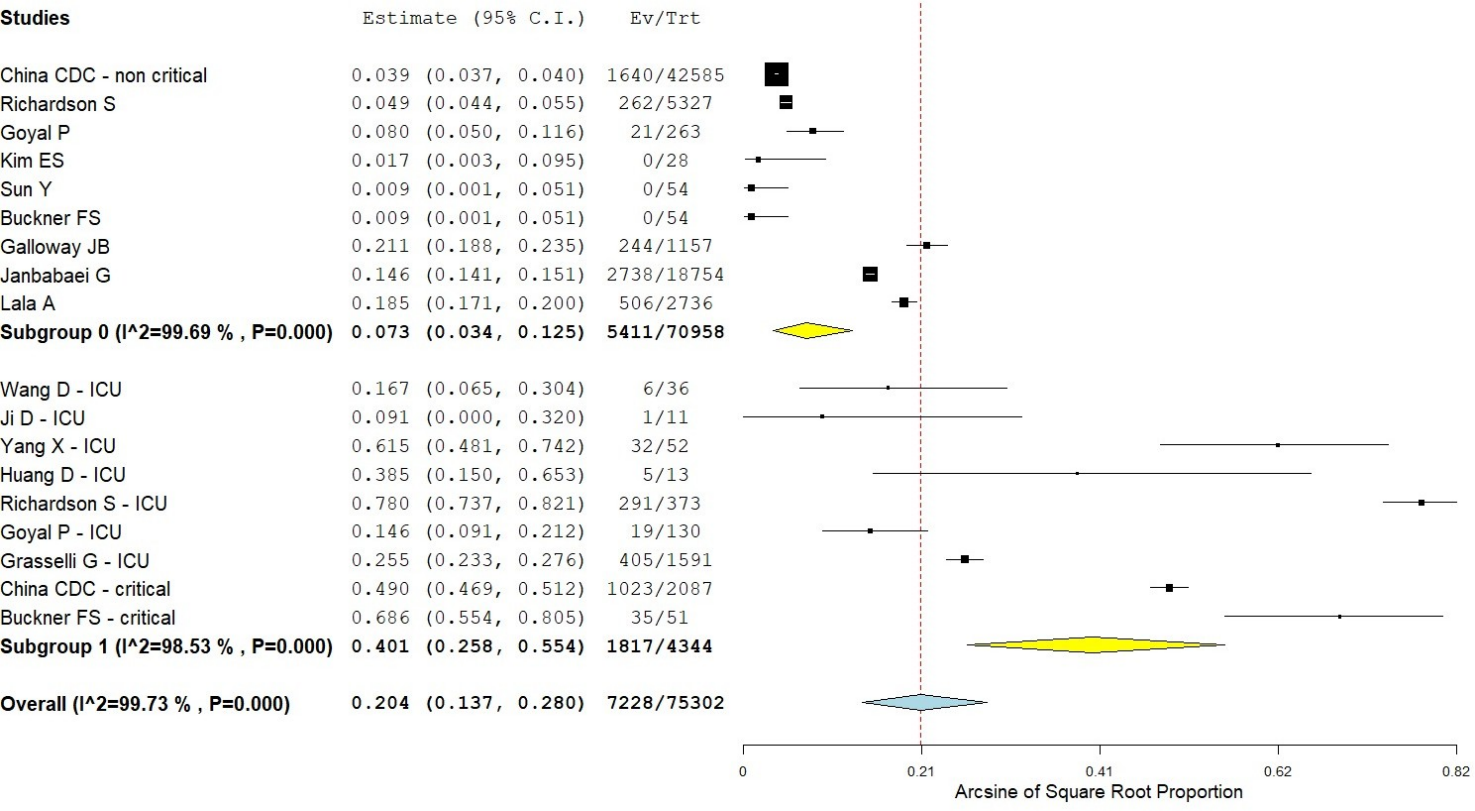
Subgroup analysis of case fatality rate. Black squares indicate the mean effect size and horizontal lines represent the 95% CI of the effect size in single studies. Diamond shapes indicate the summary effect size and the relative 95% CI for each subgroup (yellow diamonds) and the overall summary effect (light blue diamond). The red dotted vertical line indicates the overall summary effect.

Case fatality rate was 9.6% (ASRP = 0.20; 95% CI = 0.14–0.28), with large heterogeneity among the studies (I^2^ = 99.7%; Qp<0.001) (Fig 4). Subgroup analysis showed that case fatality rate was 41.8% (ASRP = 0.40; 95% CI = 0.26–0.55) for ICU and 7.6% among non-ICU patients (ASRP = 0.07; 95% CI = 0.03–0.13).

At meta-regression analysis, age (p<0.001), pre-existing cardiovascular comorbidities or risk factors (p<0.001) and the development of cardiovascular complications during COVID-19 period (p = 0.038) had a significant interaction with death (Table 4).

**Table 4:** Predictors of case fatality rate at meta-regression analysis.

Covariate	Regression Coefficient	95% Confidence Interval	P value
Age	0.006	0.003–0.008	<0.001
CV comorbidity/risk factor	0.004	0.003–0.005	<0.001
CV complications	0.001	0.000–0.003	0.038

## Discussion

This study investigates the extent of cardiac comorbidities and complications among hospitalized patients with Covid-19. In this meta-analysis, cardiovascular symptoms or complications were registered in a considerable proportion (14.1%) of hospitalized COVID-19 patients. Overall, case fatality rate was 9.6%. Not surprisingly, patients under intensive care shows highest mortality, about 6-fold compared to patients not needing intensive care. Most important, we found that both pre-existing cardiovascular comorbidities or risk factors and the development of cardiovascular complications are associated with fatality rate COVID-19 patients.

Cardiac injury is indeed common in several viral infections, for example in those caused by influenza virus. In a retrospective multicentre study, Gao et al demonstrated a high proportion (63.2%) of cardiac injury in hospitalized patients with Avian influenza A (H7N9) viral infection [22]. Retrospective studies showed that about 54% infected individuals presented cardiac injury, at the time of the H1N1 pandemic in 2009 [23]. Also, Ludwig et al, collecting data from more than 600 Americans in 2010–2012, who underwent dosage of cardiac biomarkers within one month after a laboratory-confirmed influenza infection, demonstrated elevated TNI and/or CK-MB in 143 (24%) individuals [24]. Some studies speculate about the capability of a direct viral invasion as the cause of infection-associated cardiac injury [25]. Alternatively, severe viral infections may provoke an exaggerated immune response, with releasing of various inflammatory cytokines and myocardial indirect damage [26]. In this regard, our finding of 10.34% myocardial injury rate dose not come as a surprise. Cardiac complications and/or comorbidities may contribute to mortality in Covid-19-infected patients, at least in part, because of the consequent hemodynamic deterioration.

In line with these observations, meta-regression analysis showed that both pre-existing cardiovascular comorbidities or risk factors and the development of cardiovascular complications during COVID-19, had a significant interaction with death. In agreement, previous studies have shown that during other viral infections, such as influenza epidemics, cardiac injury is strongly related to an increased mortality. Indeed, data from the severe H1N1 pandemic in 2009 showed that elevated TNI and left ventricular systolic dysfunction (EF<50%) were associated with high mortality [23]. A study by Pearce et al, conducted in Australia taking into account influenza virus type and subtype in 2007–2009, provides further evidence that severe respiratory infections may trigger the onset of cardiovascular events, implicating the influenza virus as a contributing factor [27]. Despite the primary clinical manifestation of COVID-19 is respiratory, our finding that pre-existing cardiovascular risk factors or co-morbidities are associated with both higher CV complications and fatality rate suggest that demands further investigation about the impact of cardiac involvement, in order to understand the actual incidence, the mechanisms, and outcomes of CV manifestations in COVID-19 patients.

Our results are in line with the findings of a recent review, showing that hypertension, diabetes and CVD are independent predictors of a more severe course of COVID-19 [28]. Our work confirms those findings and extend them, showing that cardiovascular risk factors and CVD are independent predictors of cardiovascular complications developed during the clinical course of the disease and death.

### Study limitations

The data reported in the studies included were collected amidst an epidemic outbreak that challenged the healthcare system. Hence, partial reporting might have happened and should be taken into account, in light of the situation. The included studies did not adopt the same criteria to define clinical endpoints. In particular, different biomarkers were used to classify myocardial injury across the studies. Notwithstanding the limitations listed above, results of this meta-analysis might be helpful for the clinical management of COVID-19 infected patients.

## Conclusions

In conclusion, results of the present meta-analysis strongly suggest that COVID-19 fatality is influenced by cardiovascular pre-existing conditions and/or cardiovascular risk factors. These findings unveil additional prognostic elements that should be taken into account, in addition to age and gender, to influence the risk prognostication and clinical management of Covid-19 patients. The association between the novel Coronavirus and cardiac complications needs further exploration and clinicians should be aware of the potential impact of cardiovascular conditions and complications in COVID-19 patients, which should require more extensive and frequent monitoring.

## Supporting information

S1 Checklist PRISMA checklist. (DOCX)

S1 Table Study Quality Assessment. Quality Assessment according to the Tool for Observational Cohort and Cross-Sectional Studies of the NIH.
(DOCX)

S1 Fig Cardiovascular comorbidities. Forest plot relative to the prevalence of any pre-existing cardiovascular comorbidity.
(DOCX)

S2 Fig Comorbidities and risk factors. Forest plots relative to the prevalence of pre-existing comorbidities and cardiovascular risk factors.
(DOCX)

S3 Fig Publication bias assessment for cardiovascular comorbidities. Funnel plot relative to the prevalence of any pre-existing cardiovascular comorbidity.
(DOCX)

S4 Fig Publication bias assessment for comorbidities and risk factors. Funnel plots relative to the prevalence of pre-existing comorbidities and cardiovascular risk factors.
(DOCX)

S5 Fig Cardiovascular complications. Forest plots relative to in-hospital cardiovascular complications.
(DOCX)

S6 Fig Publication bias assessment for cardiovascular complications. Funnel plots relative to in-hospital cardiovascular complications.
(DOCX)
