# Correction: Impact of cardiovascular risk profile on COVID-19 outcome. A meta-analysis

**DOI:** 10.1371/journal.pone.0243471

**Published:** 2020-12-03

**Authors:** Jolanda Sabatino, Salvatore De Rosa, Giovanni Di Salvo, Ciro Indolfi

An additional affiliation is missing for the fourth author. Ciro Indolfi is also affiliated with Mediterranea Cardiocentro, Naples, Italy.
