# Supplementary material for: Impact of cardiovascular risk profile on COVID-19 outcome. A meta-analysis
Source: PLoS One. 2020 Aug 14;15(8):e0237131. doi: 10.1371/journal.pone.0237131 (PMC7428172; doi:10.1371/journal.pone.0237131)
Supplement: S1 Fig — Forest plot relative to the prevalence of any pre-existing cardiovascular comorbidity. (DOCX) [file pone.0237131.s003.docx]

**S1 Fig. Cardiovascular comorbidities.**
