# Supplementary figures and images for: Impact of cardiovascular risk profile on COVID-19 outcome. A meta-analysis
Source: PLoS One. 2020 Aug 14;15(8):e0237131. doi: 10.1371/journal.pone.0237131 (PMC7428172; doi:10.1371/journal.pone.0237131)

**S2 Fig. Comorbidities and risk factors.**


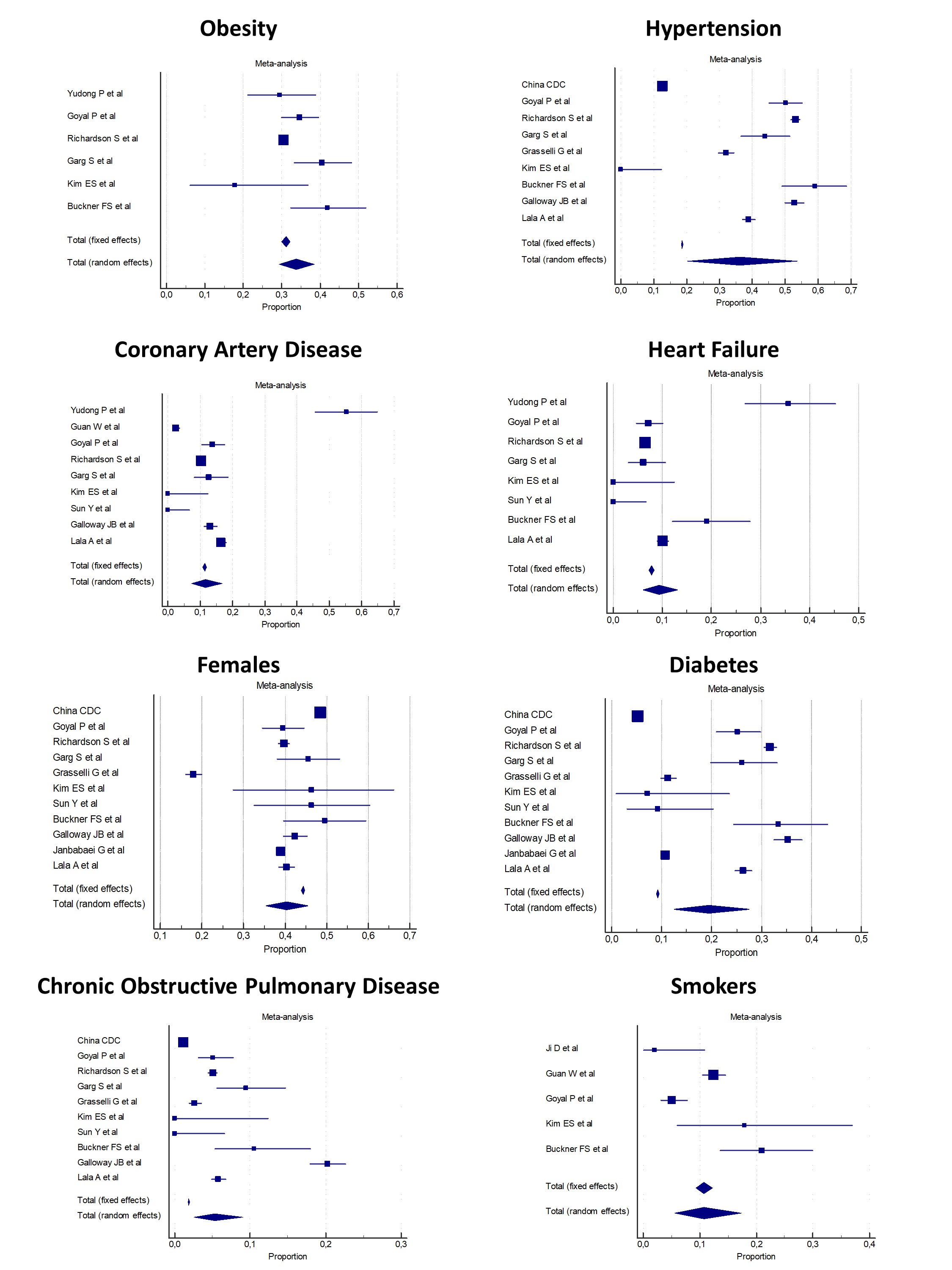

Supplement: S2 Fig — Forest plots relative to the prevalence of pre-existing comorbidities and cardiovascular risk factors. (DOCX) [file pone.0237131.s004.docx]

**S4 Fig. Publication bias assessment for Comorbidities and risk factors.**


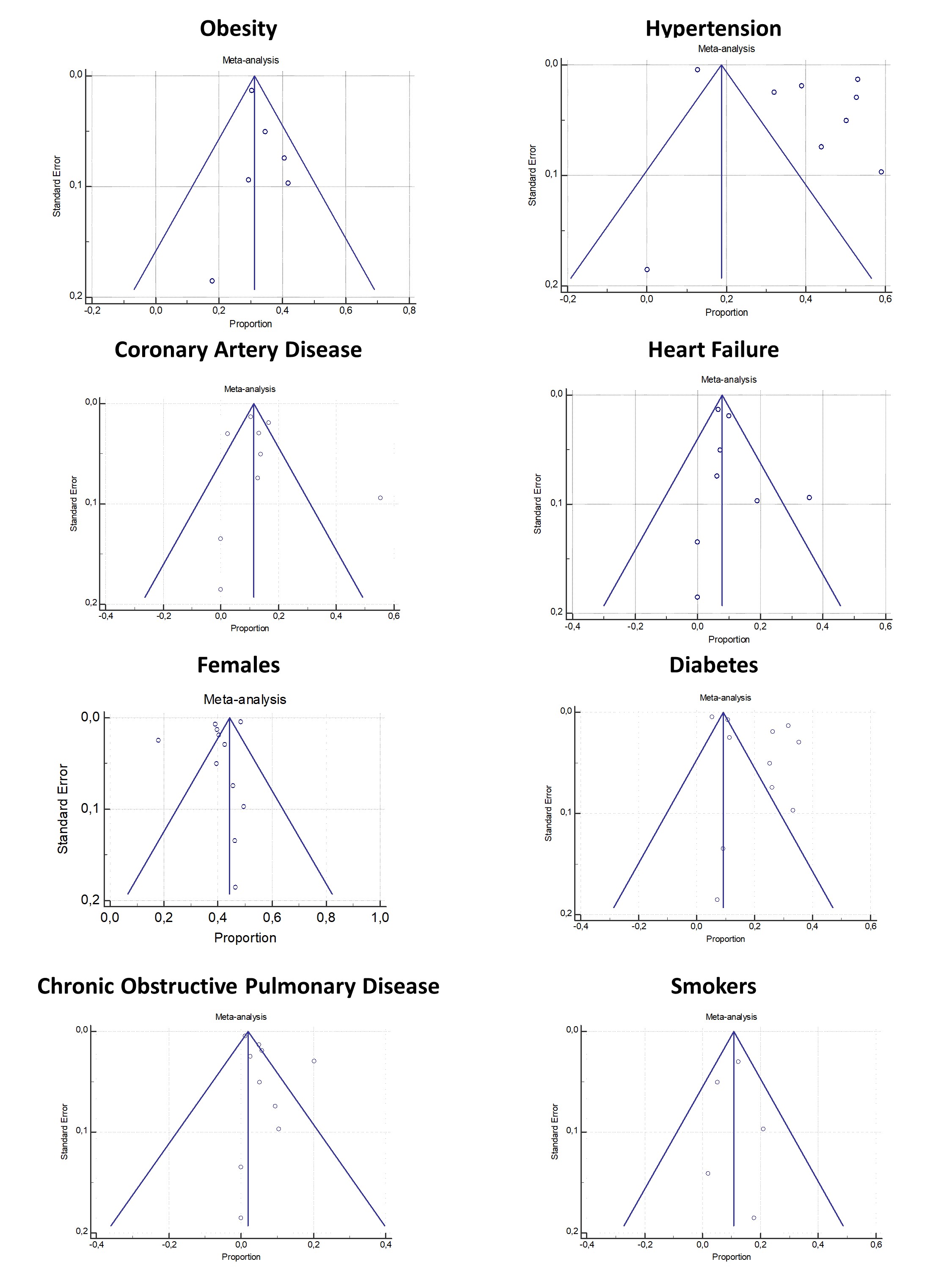

Supplement: S4 Fig — Funnel plots relative to the prevalence of pre-existing comorbidities and cardiovascular risk factors. (DOCX) [file pone.0237131.s006.docx]

**S5 Fig. Cardiovascular complications.**


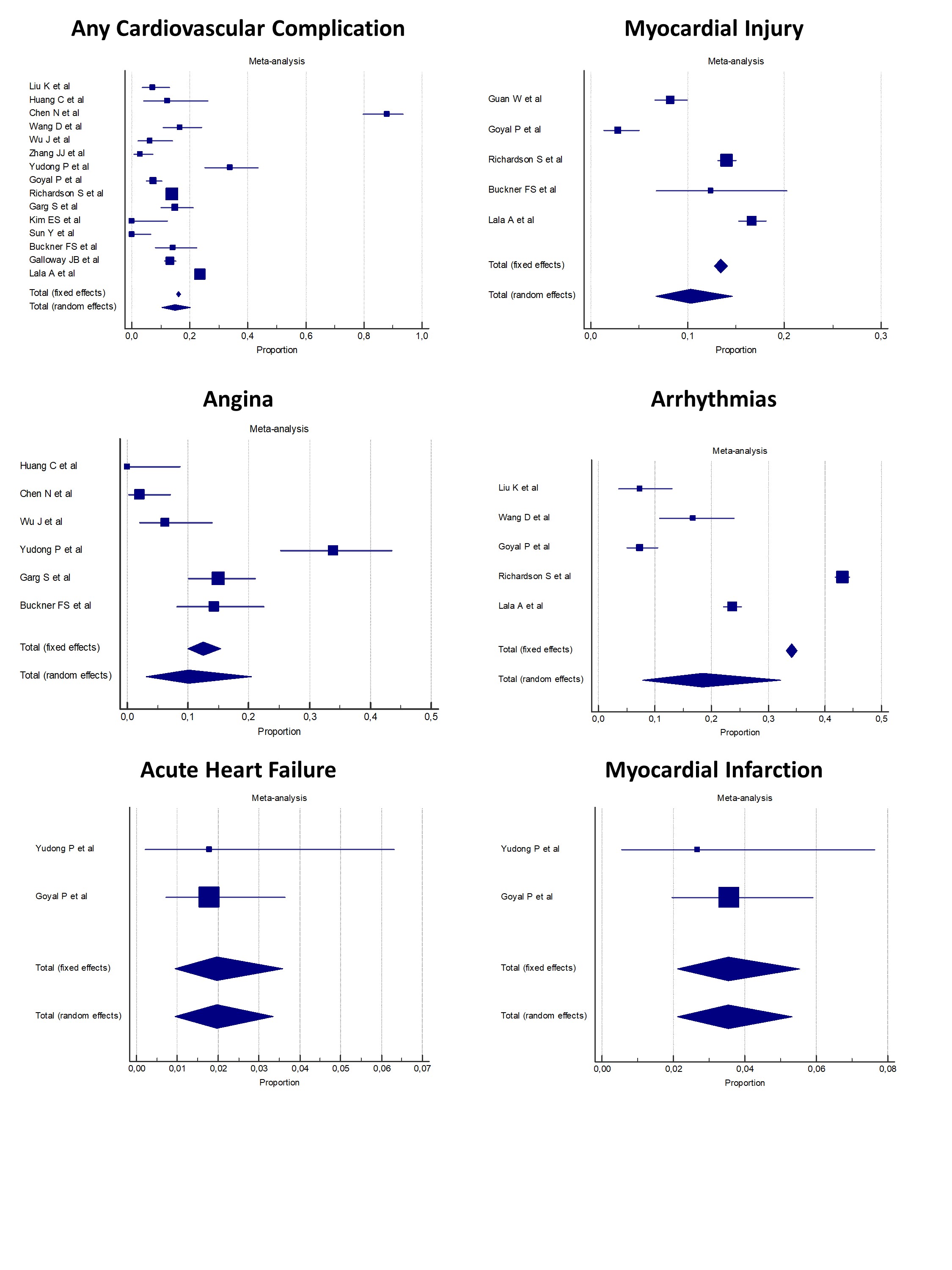

Supplement: S5 Fig — Forest plots relative to in-hospital cardiovascular complications. (DOCX) [file pone.0237131.s007.docx]

**S6 Fig. Publication bias assessment for Cardiovascular complications.**

**
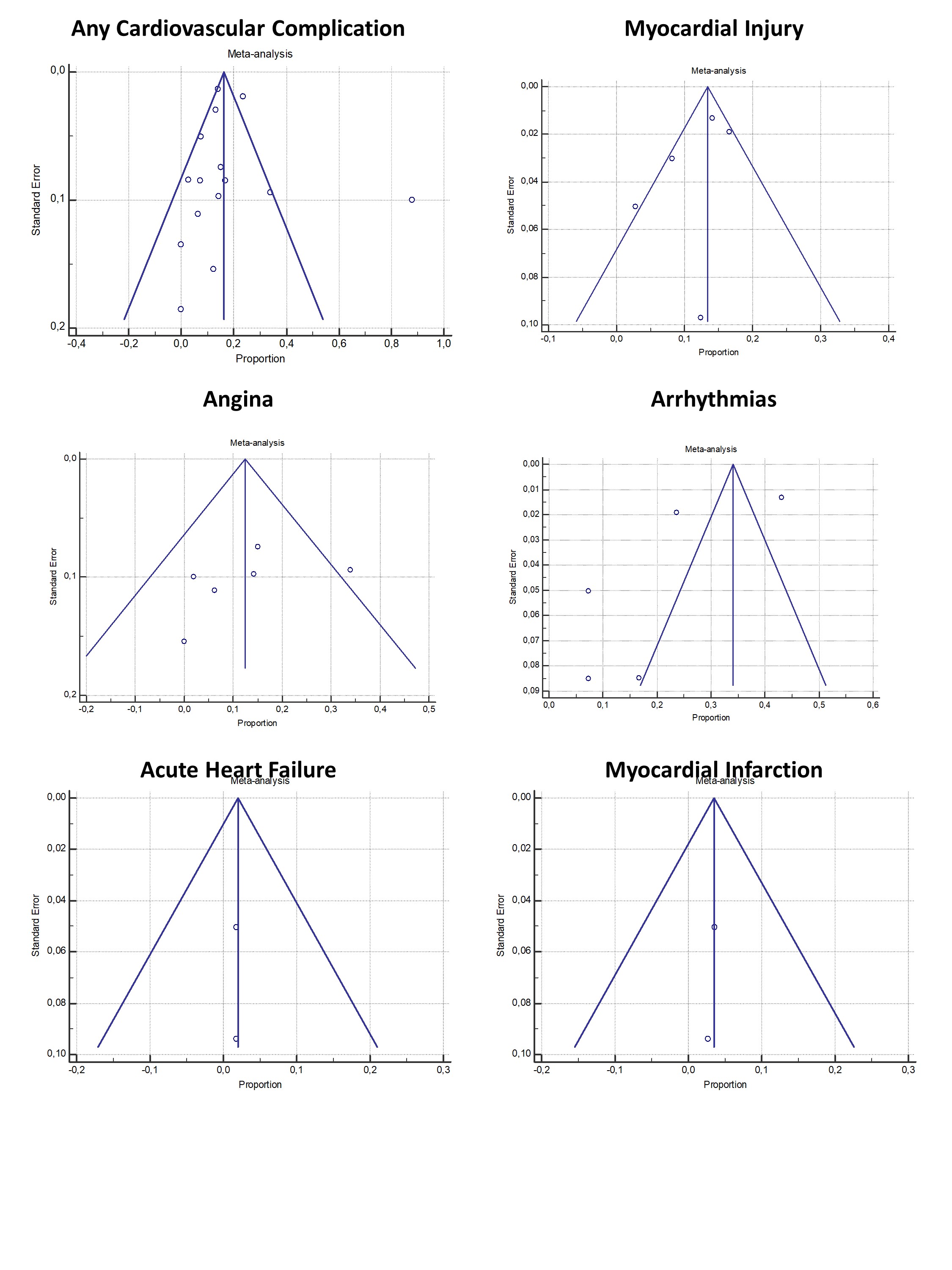
**

Supplement: S6 Fig — Funnel plots relative to in-hospital cardiovascular complications. (DOCX) [file pone.0237131.s008.docx]
